# Mechanical and radiation shielding assessment of high-density/magnetite-cementitious plaster for repair and retrofit of nuclear power plant structures

**DOI:** 10.1038/s41598-025-33014-6

**Published:** 2026-01-10

**Authors:** Eslam M. Attia, Moamen G. El-Samrah, Mohamed Rashad, Islam M. Nabil, Mohamed A. E. M. Ali

**Affiliations:** 1 Department of Civil Engineering, Military Technical College, Cairo, Egypt; 2 Department of Nuclear Engineering, Military Technical College, Cairo, Egypt; 3 Department of Physics, Faculty of Science, Fayoum University, Fayoum, Egypt

**Keywords:** Magnetite powder, Cement plaster, Mechanical properties, Radiation shielding, NPP structures, Engineering, Materials science, Physics

## Abstract

Concrete plays a critical role in nuclear power plants (NPPs) as structural material and radiation shielding. Severe defects during construction of NPP concrete could lead to catastrophic leakages. Therefore, cement plaster propably with acceptable radiation protection could be a good solution as a repairing technique. Thus, this study investigates the mechanical and radiation shielding properties of non-conventional cementitious plaster incorporating magnetite powder as a partial/full replacement for traditional sand. ​Five cement plaster mixtures were proposed with varying proportions of magnetite powder and sand, in which their mechanical and radiation attenuation properties were analyzed. Experimental evaluations including workability, density, compressive strength, and gamma-ray attenuation were investigated. Furthermore, γ-ray and fast neutron shielding has been evaluated using software programs such as EpiXS, NXCom, and MRCsC. Results showed that incorporating magnetite powder in cement plaster production lowers the compactness of the matrix by up to 46.2%. Consequently, the compressive strength was generally decreased by up to 65.4% with increased magnetite content. However, the density of the plaster was increased by up to 48.7% compared to traditional cement plaster. Furthermore, owing to increased density, the radiation shielding efficiency of magnetite cement plaster was generally enhanced. Cement plaster with 100% magnetite content achieved superior linear attenuation coefficient (LAC) for gamma rays with 264% increase at 0.01 MeV and 43% increase at 10 MeV compared to the traditional sand plaster. Also it acquired better fast neutron shielding performance, with a macroscopic removal cross-section (Σ_R_) of 0.1056 cm⁻¹ (11% increase) compared to the conventional plaster. ​This research highlights the potential of utilizing magnetite-cement plaster with enhanced radiation shielding properties for repair and maintenance strategies of NPP structures. With the achieved gamma and neutron shielding properties, such material can significantly improve safety, durability, and longevity of nuclear power plant structures.

## Introduction

Concrete is a primary structural material in nuclear power plants (NPPs) which are used for structures and biological shielding. Containment structures, a key application, are designed to withstand extreme events and act as the final barrier against radioactive release. To ensure their leak-tightness and structural integrity, age-related degradation of concrete containment buildings (CCBs) must be controlled. Issues from construction quality control and operational ageing can impair a containment’s response to accidents, reducing its structural margins. Thus, researchers aim to develop cement-based materials with enhanced radiation attenuation to enhance and repair nuclear installation structures. This involves implementing new materials with favorable absorption properties to improve durability against radiation and then ensuring safe and reliable nuclear structures^1,2^.

Therefore, many investigations have focused on concrete and other cement-based composites performance against radiation. For instance, Tousi et al.^3^ studied the effect of partial replacemet of cement with magnetite powder. The study revealed that replacing 20% of cement with magnetite improved gamma-ray shielding by up to 43%. Although magnetite can cause a slight reduction in strength compared to other mixtures, the resulting cementitious composite remains stronger than regular ones. Similarly, Barforoush et al.^4^ found that the addition of basalt fibres to high-performance concrete containing magnetite fine aggregate improved its ductility, stiffness, and radiation shielding. This mixture achieved up to a 5% increase in gamma-rays attenuation at energy levels of 1173 and 1332 keV. Moreover, Jóźwiak-Niedźwiedzka et al.^5^ investigated how the composition of concrete, specifically the type of coarse aggregate and cement, affects its durability and safety in nuclear facilities. The research focused on two key properties: gas permeability (to prevent radioactive leaks) and gamma radiation shielding. The findings showed that the choice of aggregate and cement significantly influences the concrete’s density, which in turn affects its gas permeability and radiation shielding effectiveness (measured by the half-value layer). The results are intended to help develop safer, more durable, and cost-effective concrete for nuclear constructions. Furthermore, Ghorbani et al.^6^ investigated the mechanical, fracture, and radiation shielding properties of concrete incorporating magnetite and hematite as heavyweight aggregates and nano-silica as a partial cement replacement. It was revealed that adding up to 6% of nano-silica increased fracture energy by 18.8% and 16.8% for the magnetite and hematite concretes, respectively. In terms of radiation shielding, the magnetite-based concrete demonstrated superior performance compared to the hematite-based concrete across all energy levels. This shielding advantage was further enhanced with the addition of nano-silica, as the material’s reduced porosity led to a consistent decrease in the passing radiation flux. Also, the most effective radiation attenuation was observed with a 4% addition of nano-silica.

The affected radiological and physico-mechanical due to incorporating other materials in concrete production were also illustrated. For instance, Shalbi et al.^7^ found that varying colemanite ratios in barite-concrete affect neutron and gamma shielding but reduce its mechanical strength. Only one mix of 46.95% barite and 1.85% colemanite met all performance criteria for nuclear facility use. Also, Othman et al.^8^ demonstrated that substituting aggregates with lead oxide increased concrete density, compressive strength, and gamma-ray shielding efficiency, while also improving hardness and reducing radiation intensity. Likewise, the addition of varying concentrations of tungsten oxide to heavy concrete increased the density as well as the photon shielding capability. The increase in the amount of tungsten oxide caused a reduction in the half-value layer and an increase in the radiation protection efficiency for concrete^9^. Furthermore, recent studies proved that boron carbide (B_4_C), a material renowned for its neutron attenuation properties as a fine aggregate replacement in concrete, enhanced the neutron shielding performance of concrete and other cement-based plasters^10^.

Also, Fattouh et al.^11^ explored how modifying the aggregate gradation of 100% recycled concrete aggregate affects concrete performance. It was revealed that the mechanical properties and radiation shielding significantly improved. Microstructural analysis confirmed denser cement matrices and refined interfacial zones in the recycled concrete mixes. The recycled concrete with the most adjusted gradation showed the best radiation shielding performance. Moreover, Fathy et al.^12^ evaluated the performance of high-strength concrete (HSC) incorporating two distinct nanomaterials: nano-lead monoxide (NL) and nano-granodiorite powder (NG). Each was tested separately at replacement levels up to 5% of cement weight, and an optimised hybrid mix (5% NL + 4% NG) was also developed. It was revealed that NG significantly improved compressive strength, peaking at a 17.3% increase at 4% replacement. However, NL showed a smaller strength gain of 7.96% at 3%, with strength decreasing beyond that point. Also, NL showed increased workability and delayed setting times, while NG had the opposite effect. The combined mix achieved an 11.7% strength increase and delivered superior gamma-ray shielding.

In addition to the former, as a shielding membrane for concrete structures, vast investigations considered using materials with high density to enhance radiation attenuation of cement plaster. For example, Sikora et al.^13^ investigated experimentally the effects of incorporating iron oxide nanoparticles (NPs) on the neutron and gamma-ray shielding properties of cement plaster. Cement plasters were prepared with nanomagnetite replacing of 5, 10, 15, 20, and 30% by weight of cement. The results revealed that higher Fe_3_O_4_-NP content progressively increased viscosity, yield stress, total porosity, and average pore volume, while substantially delaying early strength development and reducing 7 and 28 day compressive strength. Conversely, radiation shielding performance improved significantly, with linear increases in macroscopic cross-sections for slow, fast, and thermal neutrons, and enhanced gamma-ray attenuation across the 0.08–2.614 MeV energy range. The study showed also that Fe_3_O_4_ nanoparticles effectively produce lead-free cementitious composites with superior radiation shielding, though the associated increase in porosity and reduction in mechanical performance must be considered^13^. The results agreed with those of Kunchariyakun et al.^14^, who studied how adding nano-magnetite (nano-Fe_3_O_4_) to mortars, at concentrations ranging from 1 to 10% by weight of cement, affected their γ and neutron-radiation shielding capabilities. The results showed that the radiation-shielding properties of the mortar were improved with the addition of nano-Fe_3_O_4_ through pore filling and nucleation effects. The best physical, compressive, and radiation shielding properties were achieved with a 5% addition of nano-Fe_3_O_4_, which increased the material’s photon linear attenuation coefficient from 0.144 cm^−1^ to 0.155 cm^−1^. The compressive strength was 216.79 ± 6.19 ksc at 28 days. The neutrons’ fast neutron removal cross-section (Σ_R_) increased from 0.1109 cm^−1^ in the control sample to 0.1192 cm^−1^ with the addition of 5% nano-Fe_3_O_4_. Based on these findings, nano-Fe_3_O_4_ added to mortar may reduce the effects of radioactive waste on people and the environment by attenuating gamma rays instead of neutrons. This could pave the way for new radiation-shielding materials for radioactive storage facilities^14^. While, in a different approach, Ali et al.^15^ studied experimentally the effects of implementing sustainable powders on radiation shielding parameters of cement plaster. It was concluded that the addition of nano-titanium to conventional plaster led to superior enhancement in the compressive strength relative to traditional plaster. Conversely, fully replacing conventional silica sand with powdery materials - steel slag, hematite, and bentonite - generally reduced the compressive strength of cementitious plasters. However, the radiation shielding properties have been enhanced by up to 26%.

In summary, although there have been extensive investigations on the radiation shielding properties of concrete and other cement-based composites. However, there is a lack of information regarding the radiation shielding chracteristics of cement plaster produced with magnetite powder as a sustainable replacer/contender for traditional sand. Such plaster can be easily formed and used for repairing structures in nuclear applications. During the construction of nuclear power plant projects, there are some structural elements with radiation protection properties such as the reinforced concrete walls of the reactor core cavity or spent fuel pools. It was revealed that there are always common mistakes and defects during construction of such concrete elements (e.g. honey- combs, hair cracks, segregation, etc.). Therefore, cementitious plasters propably with acceptable radiation protection must be used in such repairing applications. Also, the design of cement plasters’ radiation shields should be appropriate for the integration of these reinforced concrete elements.

Therefore, the aim of this research is to present a sustainable cement plaster mixtures, produced with nationaly available magnetite powder, probably with proper static performance and radiation shielding capabilities. Such plasters extremly necessary for structural elements that develop defects either during construction or after the operation of nuclear power plants.

## Materials and methods

### Materials

Portland Cement CEM-I was utilized in this study. Its physical properties is presented in Table 1 while, its chemical composition determined via X-Ray Fluorescence (XRF) analysis, is demonstrated in Table 2.

**Table 1:** Physical properties of utilized cement.

Item	Unit	Test Result
Fineness, Blaine	cm^2^/gm	3550
Initial Setting Time	Minutes	170
Soundness Expansion	Mm	1
Water consistency	%	27
Loss on ignition	%	1.6
Particle size	microns	15

Data are presented as median (interquartile range). * Comparison of the differences between AFC_3D_ and AFC_2D_, Significance is indicated by *P* < 0.05 (Wilcoxon signed-rank test). AFC, antral follicle count; AMH, anti-Müllerian hormone.

**Table 2:** Chemical composition of utilized cement.

Oxides	Test Result %
SiO_2_	20.55
Al_2_O_3_	5.58
Fe_2_O_3_	4.33
CaO	58.91
MgO	1.33
SO_3_	3.39
Na_2_O	0.45
K_2_O	0.25

**Fig. 1:**
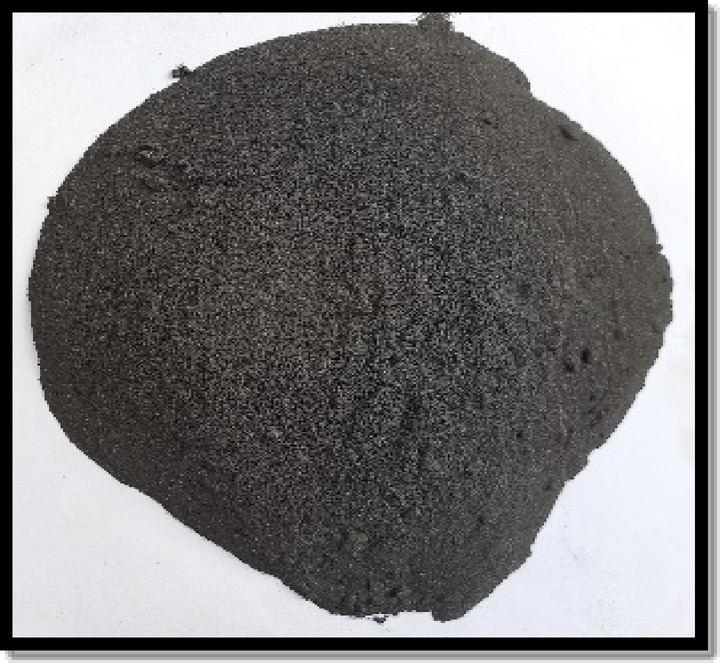
Utilized magnetite powder.

Also, natural siliceous sand as fine aggregate with specific gravity of 2.65, maximum nominal size of 0.2 mm and water absorption% of 0.4, in accordance with ASTM D75^16^ was used in this research work. Moreover, nationally available magnetite powder (shown in Fig. 1) with specific gravity of 5.1 was implemented in cement plaster production. The utilized magnetite powder has an average particle size of 825 μm and 5.17 gm/cm^3^ real density.

**Fig. 2:**
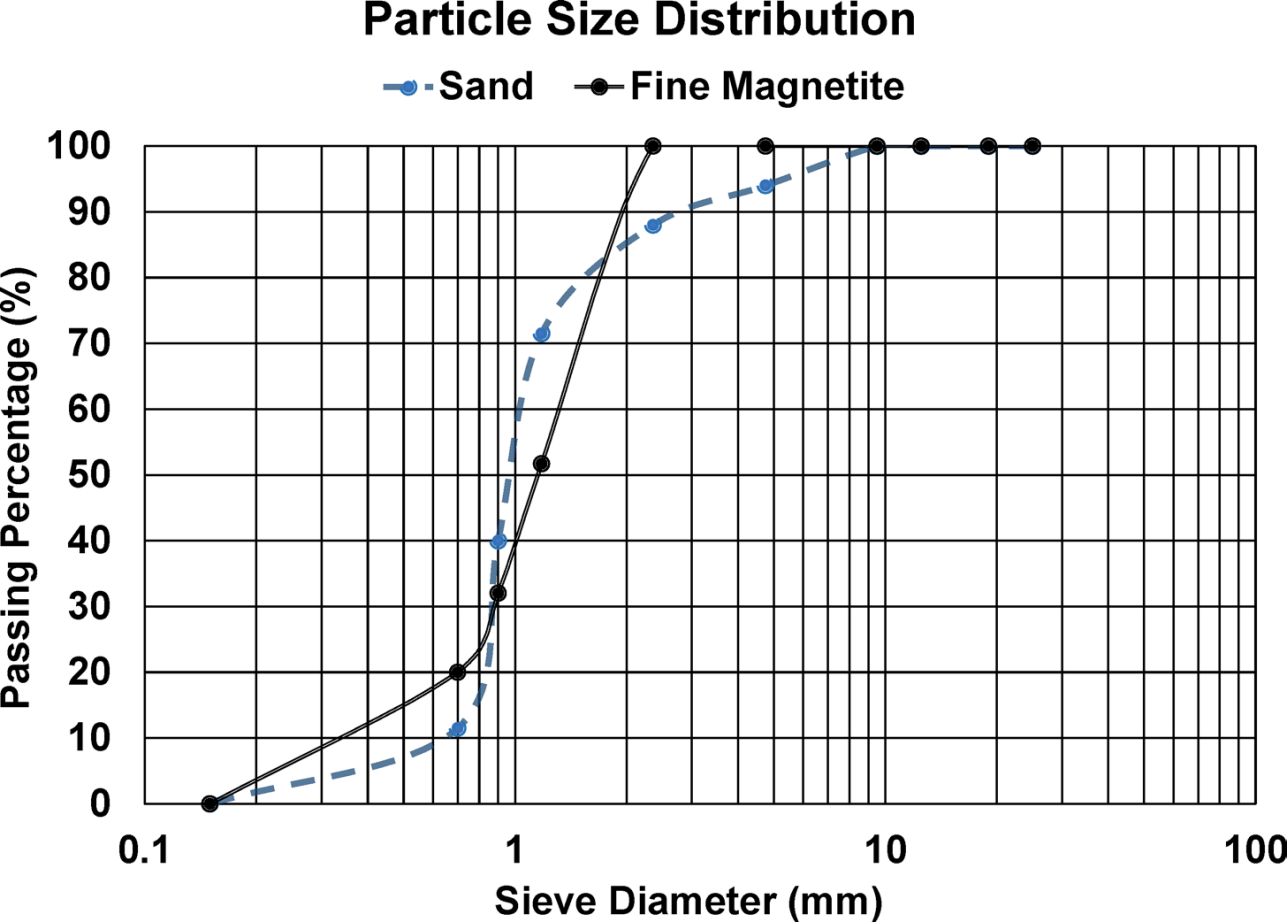
Particle size distribution of utilized sand and magnetite powder.

The idea behind utilizing magnetite powder in this study as a contender for traditional silica sand is due to its high abundance, low price, high content of iron and possibility of use without further processing, which makes it a suitable option for making cementitious composites that protect against radiation^17–19^. The analyzed chemical composition via X-Ray Fluorescence (XRF) of the utilized magnetite powder is presented in Table 3, while Fig. 2 demonstrates the sieve analysis distribution of the utilized sand and magnetite powder.

**Table 3:** Chemical composition of magnetite powder.

Oxide type	Result %
Na_2_O	0.3
MgO	0.8
Al_2_O_3_	2.6
SiO_2_	8.17
P_2_O_5_	0.12
SO_3_	0.01
K_2_O	0.3
CaO	2.12
TiO_2_	18.8
MnO	0.4
Fe_2_O_3_	63.3
ZrO_2_	0.11
PbO	0.51
Bi_2_O_3_	0.44
LOI	1.21

### Design of the proposed cement plaster mixtures

This experimental research develops non-conventional cement plaster mixtures with magnetite powder as a partial replacement for traditional silica sand. Then, it assesses the mechanical and radiation shielding characteristics of proposed mixtures. The cement plaster mixtures were formulated in accordance with the ACI 211.1^20^ guidelines, which provide a flexible framework for concrete mix design. Although this standard was originally intended for concrete, its guidelines can be adapted for cement plaster through a series of steps aimed at achieving optimal strength and workability. As per the guidelines, a volumetric ratio of 1:3 to 1:4 (cement to fine aggregates) is typically recommended for cement plaster design. In this study, a 1:3 ratio was employed to achieve the proper strength.

Five different cement plaster mixtures were prepared from silica sand and magnetite powder (a mineral whose primary component is iron oxide). The proposed mix constituents, including silica sand, magnetite powder, and water, were presented as a ratio to cement content in the mixtures in Table 4.

**Table 4:** Proposed cement plaster constituent proportions.

Content/ Mix ID	Cement	Sand	Magnetite Powder	Water
NP	1	3	–	0.5
MP25	1	2.25	0.75	0.5
MP50	1	1.5	1.5	0.5
MP75	1	0.75	2.25	0.5
MP100	1	–	3	0.5

### Preparation, mixing and curing

**Fig. 3:**
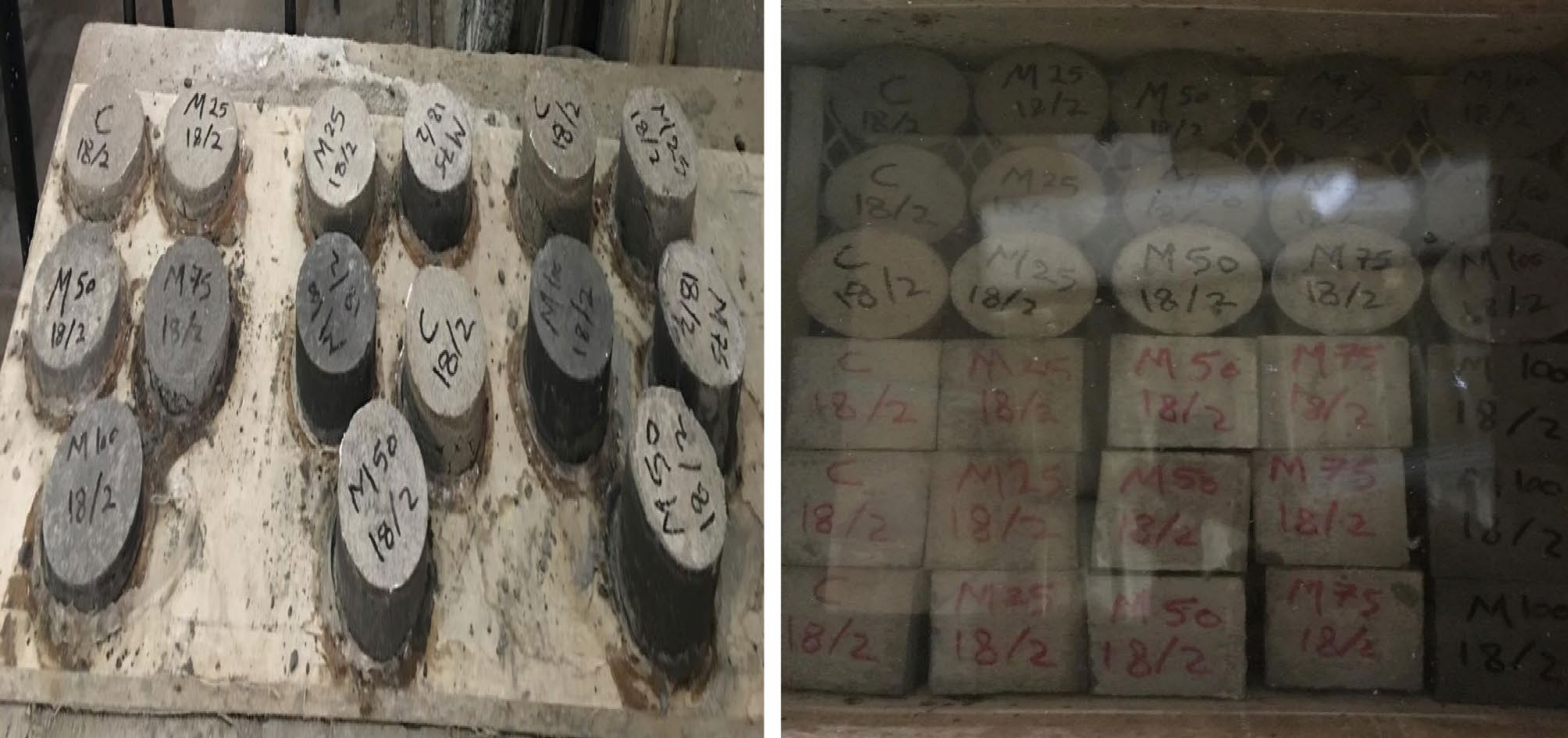
Production and curing of test specimens.

All cement plaster ingredients were firstly prepared and weighted for each mixture. A step by step mixing technique was followed to acheive adequate consistency. For instance, the silica sand and magnetite powder underwent dry mixing for one minute to promote uniform dispersion and prevent agglomeration. The uniform dispersion of magnetite powder through the mixtures led to convert the grey colored traditional cement plaster (NP) into darker/black colored mixtures based on the magnetite content within the mixture as shown in Fig. 3. Thereafter, cement was then incorporated and mixed for two additional minutes to achieve comprehensive integration and effective blending with dry fine constituents. The dry constituents of the proposed plaster mixtures were blended using a concrete mixer to ensure uniform distribution and minimize segregation. Then, pre-measured quantities of water were subsequently added to complete the mixture. Finally, the fresh plaster was poured into moulds and compacted using a mechanical vibrator to eliminate any entrapped air, thereby ensuring a dense and homogeneous structure. The upper surface of specimens was smoothed using a trowel to ensure geometric uniformity, which is essential for consistent testing outcomes. After 24 h, the specimens were demoulded and subsequently immersed in a water tank as shown in Fig. 3. They were maintained at a stable temperature of 25 ± 2 °C and a relative humidity exceeding 95%. This controlled environment provided ideal conditions for the hydration process and strength development by mitigating moisture loss and temperature variability. Specimens remained submerged until their respective testing intervals to ensure adequate curing and optimal mechanical performance.

## Testing

The tested specimens in this research work have two different shapes: (a) standard cubes (100 mm × 100 mm × 100 mm) and (b) cylinders with a standard diameter of 100 mm and different thicknesses of 20 mm, 40 mm, and 60 mm. The cubes were prepared and tested to measure compressive strength and density, while the cylinders were prepared and tested to evaluate radiation shielding characteristics against gamma radiation. Three identical specimens were prepared from each mixture as the measurements were performed on identical triplicates, resulting in 6 cubes and 12 cylinders per mix design. In total, 30 cubes and 60 cyinders were cast and tested in this research to provide a comprehensive dataset for evaluating the mechanical characteristics and shielding behavior of the proposed cement plasters with varying thicknesses.

### Workability test

**Fig. 4:**
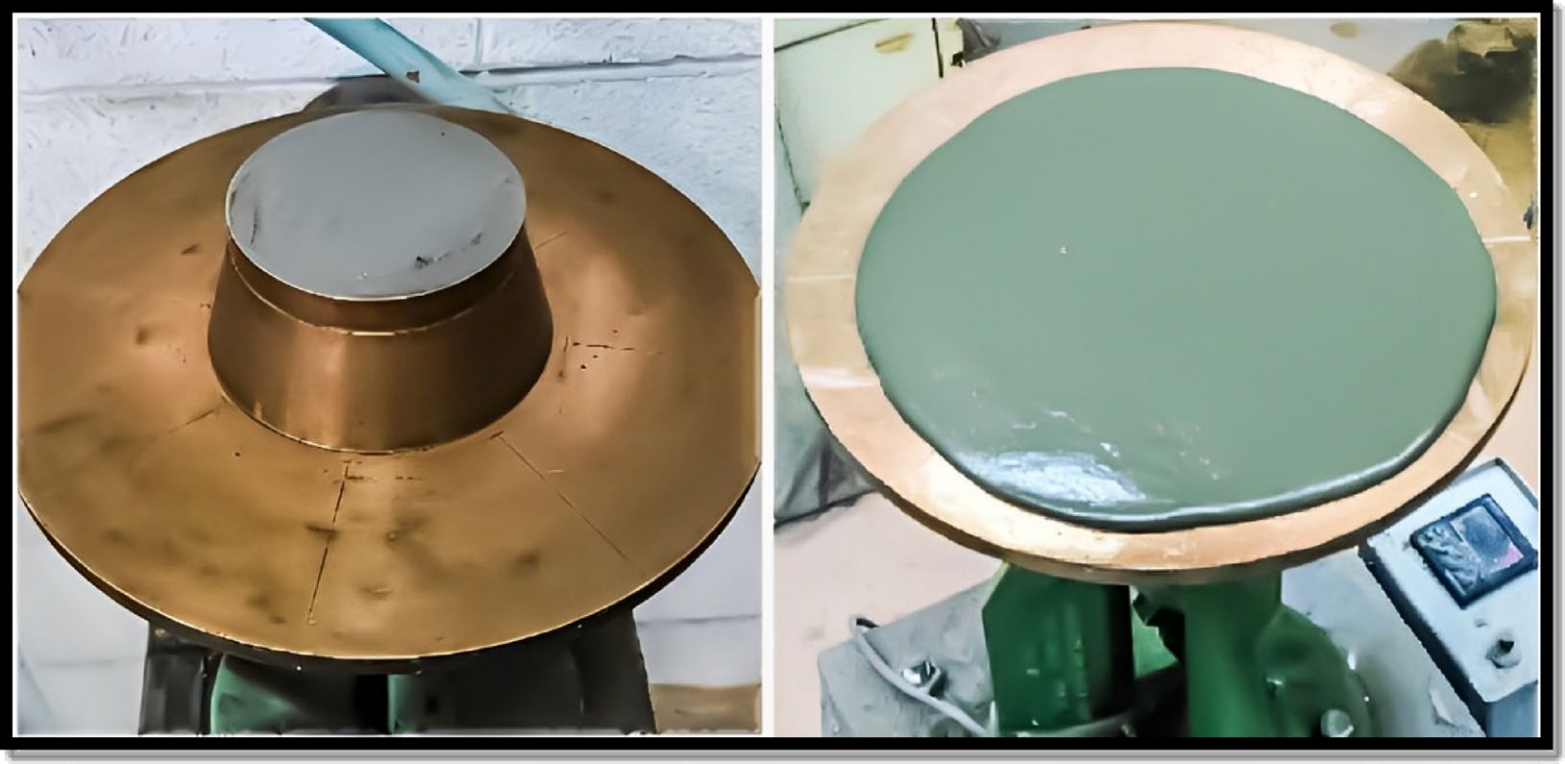
Applied fow table test.

Flow table tests were applied in this study, as presented in Fig. 4, to evaluate the effect of magnetite powder incorporation on the flowability of freshly mixed cement plaster mixtures as per the guidelines of ASTM C230 (Standard Specification for Flow Table for Use in Tests of Hydraulic Cement)^21^.

### Density measurments

The density of different cement plaster mixtures was determined by measuring the mass of a standard cube of cement plaster using a calibrated scale divided by its volume. This approach ensures precise density measurements, which are crucial for radiation shielding analytical assessments.

### Compressive strength test

**Fig. 5:**
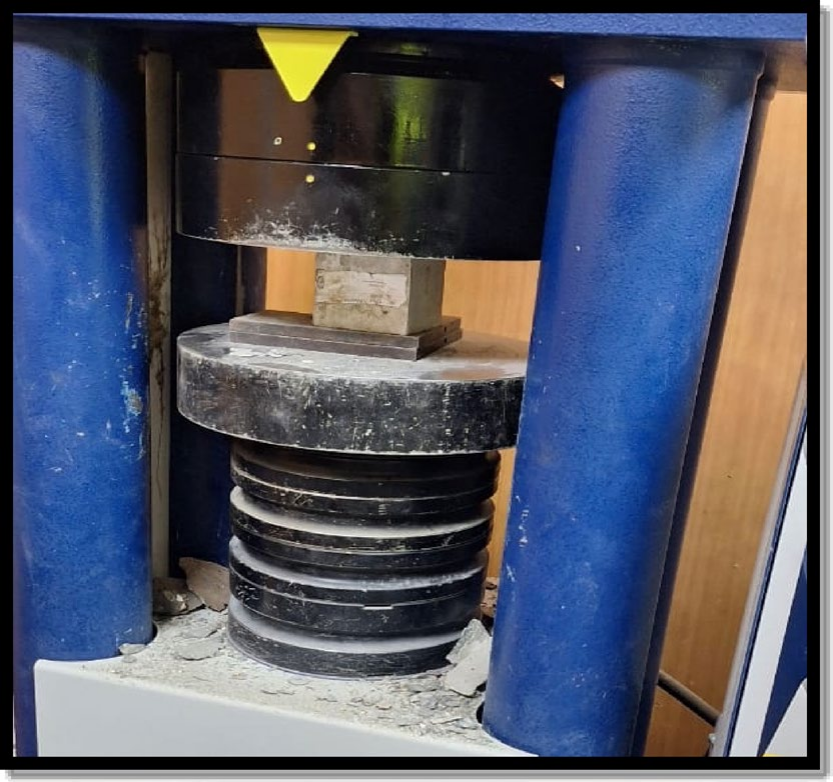
Compressive strength testing technique

In this study, the compressive strength of various cement plaster mixtures was determined according to ASTM C39 guidelines^22^. The testing equipment used for the aforementioned test was a 2000 KN universal testing machine as shown in Fig. 5. The test was performed using a set of three cubic specimens (100 mm × 100 mm × 100 mm) for each cement plaster mixture at curing ages of 7 and 28 days.

### Radiation shielding experimental testing

**Fig. 6:**
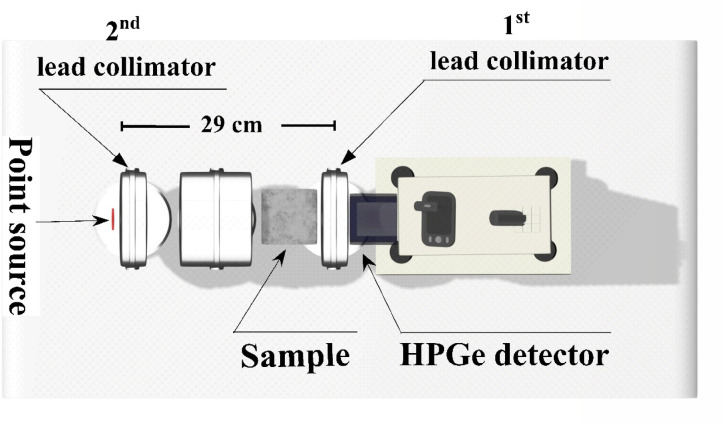
Experimental setup of gamma rays attenuation measurements.

Cylindrical specimens of 100 mm diameter and varying thicknesses were prepared for each cement plaster mix with age of 28 days and tested at specific γ-radiation energies. The examined γ-rays energies along with their radioactive sources are; 0.662 MeV for Cesium (Cs-137), and 1.173 and 1.332 MeV for cobalt (Co-60) sources, respectively (Fig. 6). The utilized detector through the attenuation measurements is HPGe semiconductor detector linked to a multichannel analyzer with Genie-2000 software (version 1.1.2, Canberra 2010). After subtracting the background radiation, the γ-rays that were emitted by sources without a shield and by uncollided beams that passed through various sample thicknesses were all recorded. An additional 100 × 100 mm^2^ lead collimator was used to shield the detector from scattered gamma rays and background radiation, allowing for precise measurements, and a 100 × 100 mm^2^ lead holder (source collimator) with a 3 mm diameter aperture was used to secure the gamma radioactive source^27^. A horizontal straight alignment of the experimental setup components was used in conjunction with collimators to achieve accurate shielding parameters for the studied mixtures. This was done solely to maintain a narrow beam geometry and ignore build-up factors. Each measurement was repeated three times and the average has been taken as the representative value while the standard deviation has been considered as the indicator for the uncertinity. A fixed distance of 290 mm was maintained between the source and the front face of the detector, as shown in Fig. 6, and the gamma-rays beam, samples, and detector were all positioned on a horizontal plane. In order to obtain transmission curves, the intensity of uncollided γ-rays that passed through the material at different thicknesses was evaluated using the Beer-Lambert Equatio^23,24^.

Then, the linear attenuation coefficient (µ) at the studied energy for each sample could be calculated based on the following Beer–Lambert Eqs^25,26^.

1 $${\text{I}}_{{\text{x}}} = {\text{I}}_{{\text{o}}} e^{-\upmu x}$$

where (I_x_) is the intensity of transmitted γ-photons through the material after subtracting any counts due to the background radiation, (I_o_) is the intensity of the primary γ-rays emitted from the source after subtracting any counts due to the background radiation, (x) is the attenuator thickness, and (µ) is the linear attenuation coefficient. In order to minimize the primary radiation to half (HVL) or tenth (TVL) of its initial value and determine the necessary shield thickness to achieve safe doses^25–27^.The following equations can be used to calculate both shielding thicknesses as follows^27^:

2 $$HVL=\frac{\text{L}\text{n}2}{{\upmu}}$$

3 $$TVL=\frac{\text{L}\text{n}10}{{\upmu}}$$

### Analytical assessment of radiation shielding

The radiation shielding analytical assessment was performed through this study in order to verify the experimental investigation then to proceed further with the study to assess the shielding capabilities of the prepared cement plaster mixes at wide range of energies. Some of the most significant shielding parameters, including the linear attenuation coefficient (µ), the effective atomic number (Z_eff_), and the macroscopic fast neutrons removal cross-section (Σ_R_), were obtained through analytical means by utilizing dependable software tools. These tools rely on validated analytical and semi-empirical models, cross-sectional databases, and/or rigid simulation engines to compute the necessary shielding parameters. Examples of such tools include EpiXS^28^, NXCom^29^, MRCsC^30^. One of the most important aspects of performing these analytical calculations employing such reliable software tools is the assessment during the initial design phase of the shield. Also another privilege is the possibility to expand the study at wide range of energies that can’t be experimentally done easily due to the lack of radioactive sources that can cover all these energies beside the high costs and prolonged testing time^31,32^.

The γ-rays shielding properties of the prepared samples were evaluated using EpiXS software which is a Windows-based interpolation software employing the EPICS2017 cross-sectional database^33^. This user-friendly software, available for download from the Philippine Nuclear Research Institute, calculates various photon shielding parameters, including linear attenuation coefficient (LAC), mass attenuation coefficient (MAC), half-value layer (HVL), tenth-value layer (TVL), mean free path (MFP), effective atomic number (Zeff), and buildup factors (e.g., energy absorption buildup factor (EABF) and exposure buildup factor (EBF)). The inputs needed are simply the chemical compositions of the shield understudy and its density^33^. On the other hand, for fast neutrons removal assessment, both NXCom^29^ and MRCsC^30^ softwares were employed as the former tool can set the lower limit and the latter one can set the upper limit for a range through which the accurate value of the macroscopic fast neutron removal cross-section (Σ_R_) should lie^19,23,26^. Both programs need only the composition of the shield and its corresponding density to calculate the characteristic (Σ_R_) employing an equation depends on a linear summation method as follows^30^ ;

4 $$\:\varSigma\:R={\sum\:}_{1}^{n}{\uprho\:}\text{s}\text{w}\text{i}\left(\frac{\varSigma\:R}{{\uprho\:}}\right)i={\sum\:}_{1}^{n}{\uprho\:}\text{i}\left(\frac{\varSigma\:R}{{\uprho\:}}\right)i$$

Where; ρ_s_ is the shield density (g/cm^3^), w_i_ and (Σ_R_/ρ)_i_ are the weight fraction and mass removal cross-section of the i^th^ element, respectively.

## Experimental results

### Workability

**Fig. 7:**
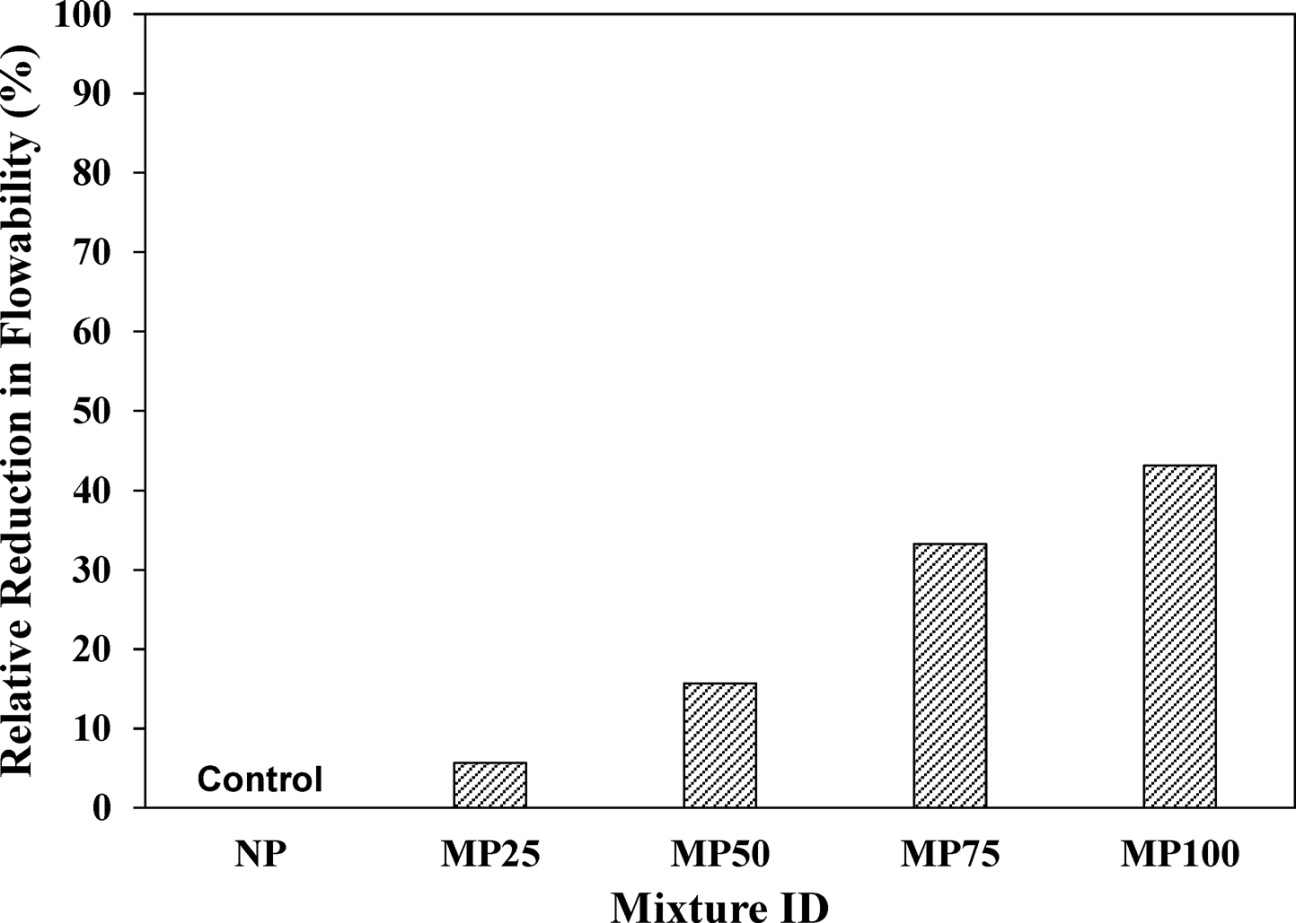
Relative reduction in flowability of different cement plaster mixtures.

The relative reduction in flow diameter for the different cement plaster mixtures relative to that of the normal cement plaster (NP / control) specimen is presented in Fig. 7. The flowability tests showed that the control (NP) mixture could flow under its self-weight, while the replacement of silica sand with magnetite powder (partially or totally) led to an overall reduction in workability as shown in the figure. For instance, the flow of the mixture MP25, which has partial replacement of silica sand by 25% of magnetite powder, was decreased by about 5.8% compared to that of the control NP mixture. Similarly, the flowability decreased by about 16.5%, 32.6% and 46.2% due to 50%, 75% and 100% magnetite powder replacement, respectively, compared to that of the NP mixture, which was made with silica sand alone. This reduction in flowability could be attributed to the incorporation of fine magnetite powder, which increases the specific surface area of the mixtures, consequently increasing their water demand, which may lead to reduced workability of the fresh matrix. It should be noted also that the reduction in flowability can affect the compactness of the matrix, acting as an exogenous parameter, which generally affects the mechanical performance.

### Density

**Fig. 8:**
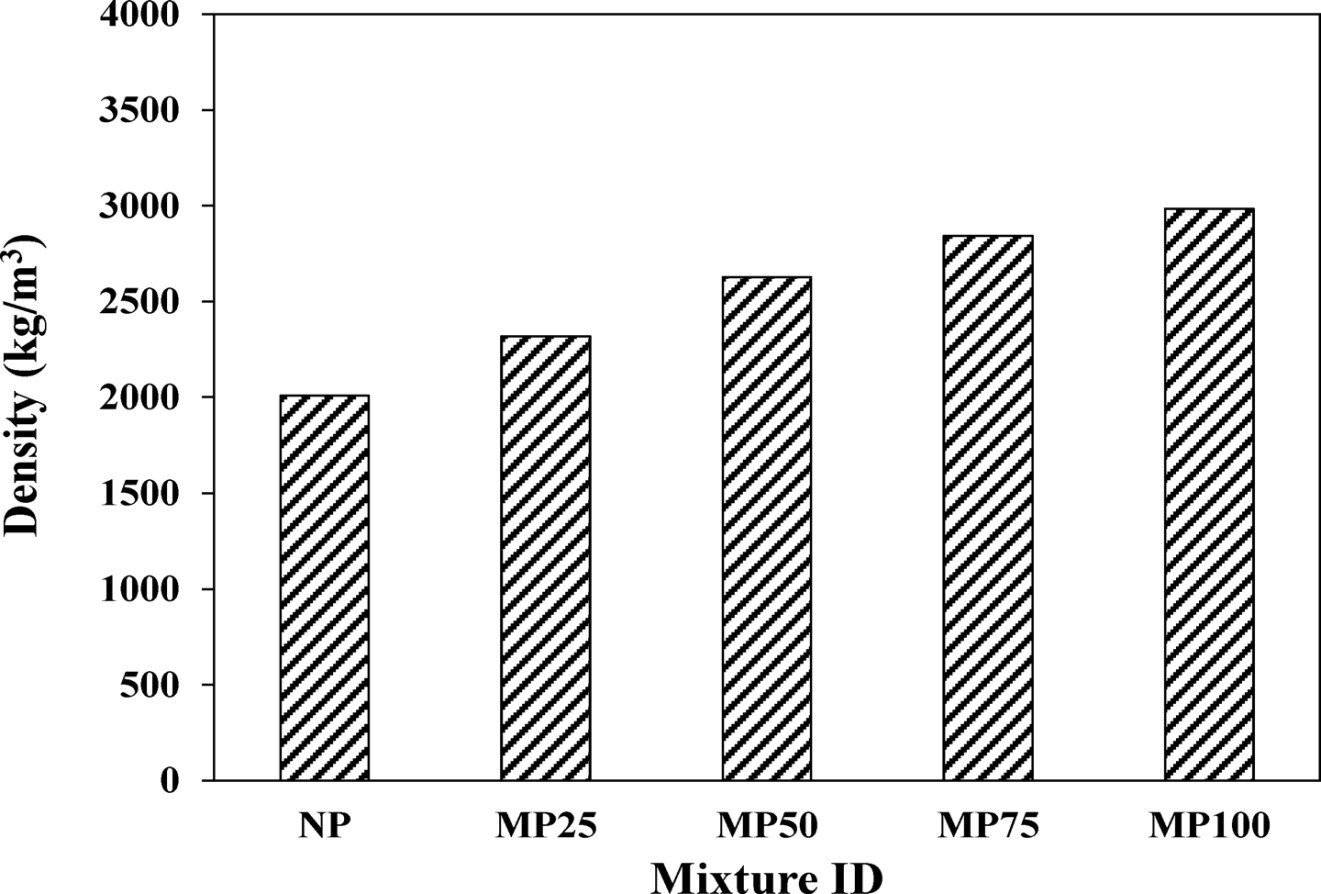
Density of various cement plaster mixtures.

Figure 8 illustrates the density of different cement plaster mixtures incorporating silica sand and/or magnetite powder. It was revealed that all magnetite powder mixtures achieved higher density compared to the control mixture, which was produced with silica sand only, as shown in the figure. For example, the implementation of magnetite powder by 25%, 50%, 75%, and 100% replacement with silica sand increased the density of the cement matrix by about 15.5%, 30.9%, 41.6%, and 48.7%, respectively, compared to that of the NP control mixture. This could be attributed to the incorporation of magnetite powder in cement plaster production, which is a black obaque mineral with a metallic lustre, a high specific gravity of 5.1 (twice that of normal silica sand), a high surface area and micro-filling capability.

### Compressive strength

**Fig. 9:**
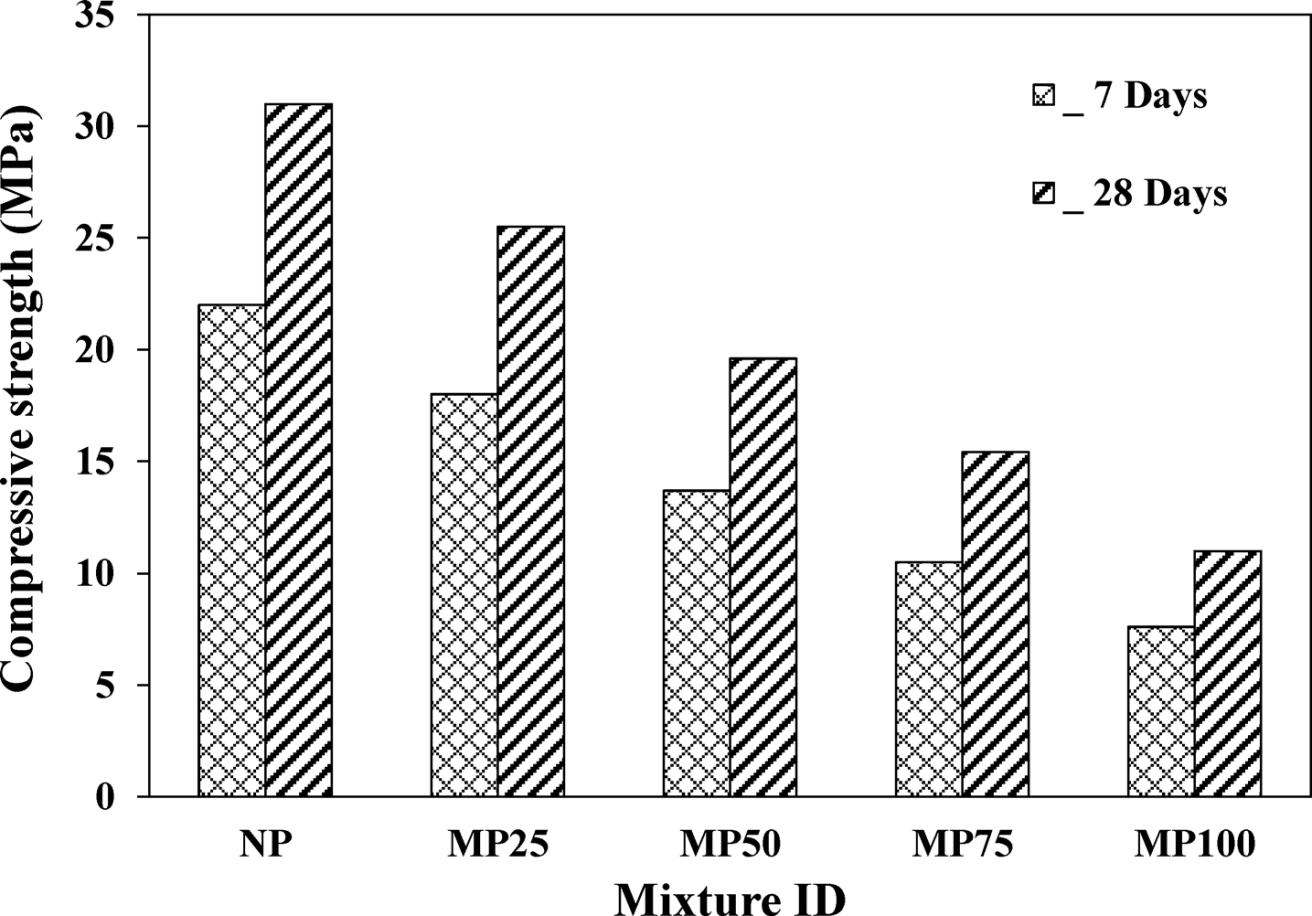
Compressive strength of different cement plaster mixtures at 7 and 28 days.

The compressive strength test was applied on the different cement plaster specimens at 7 and 28 days of maturity. Figure 9 displays the variation in compressive strength test results at different ages. As shown in the figure, the achieved compressive strength at 28 days was generally higher than that obtained at 7 days for all mixtures. This behavior is attributed to the formation of hydration products and domination of hydrated calcium silicate (C-S-H gel) among these hydration products. Also, it was revealed that the implementation of magnetite powder in cement plaster production generally reduced the compressive strength of the mixtures at all testing ages, as illustrated in the figure. For instance, at the testing age of 7 days, the compressive strength of cement plaster mixtures incorporating 25%, 50%, 75%, and 100% magnetite powder as a partial replacement of silica sand decreased by about 18.2%, 37.7%, 52.3%, and 65.4% relative to that of the normal cement plaster mixture (control). Similarly, the compressive strength of MP25, MP50, MP75, and MP100 was reduced by about 17.7%, 36.8%, 50.9%, and 64.5% compared to the NP control matrix at 28 days of maturity. The general reduction in compressive strength of cement plaster could be attributed, based on the abovementioned discussion, to the workability reduction (due to the implementation of fine magnetite powder, which increases the specific surface area of the mixtures), which can lower the compactness of cement plaster, thus resulting in a dramatic reduction in the compressive strength of the matrix. Similar conclusions were drawn elsewere^15^. Based on the aforementioned results and following the ACI 318 standard, incorporating magnetite powder by up to 100% as a replacement for conventional sand still achieved the minimum requirements of cement plasters’ compressive strength for non-load-bearing radiation shielding layers (non-structural applications).

### Experimental results of gamma rays’ shielding

**Fig. 10:**
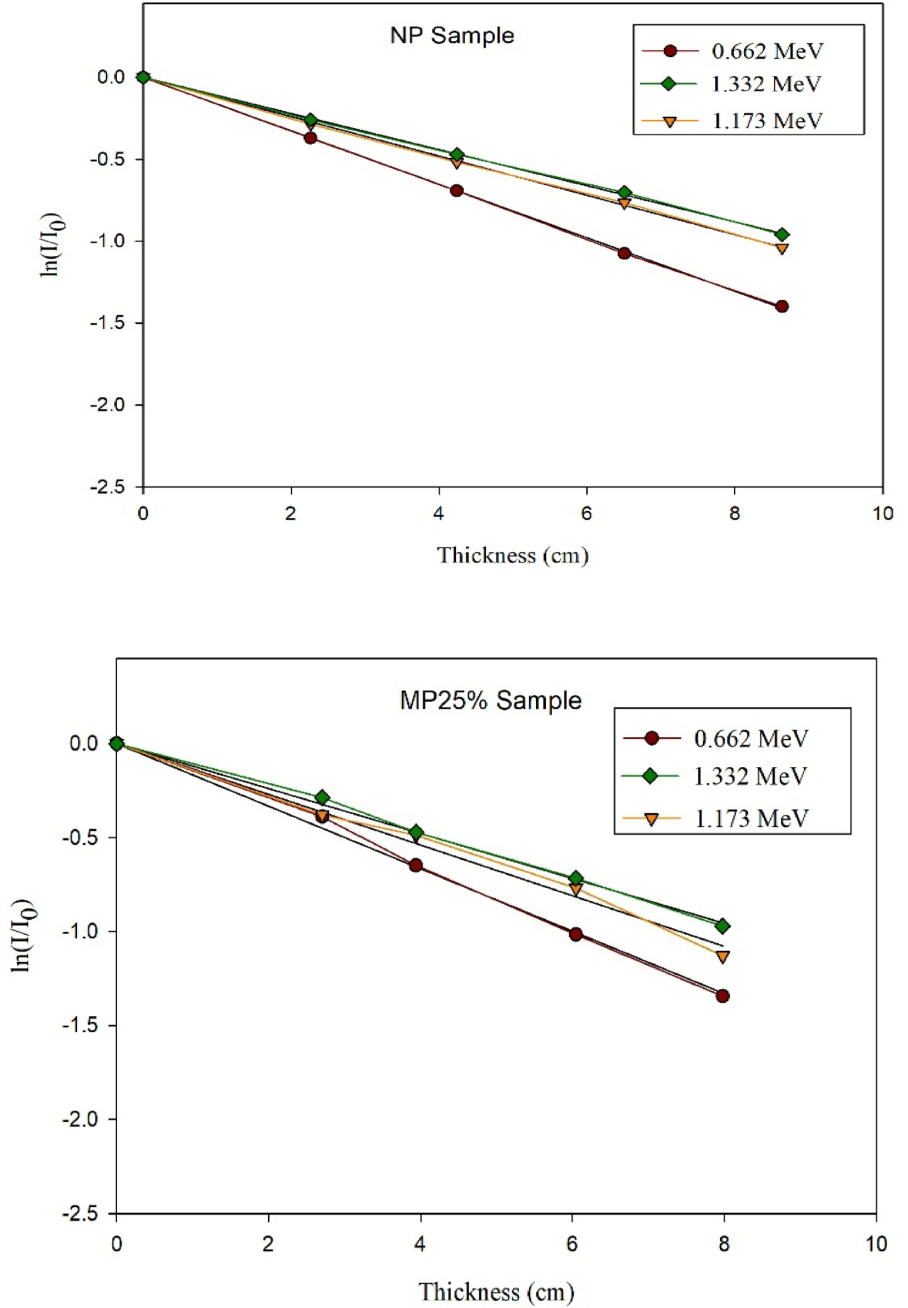

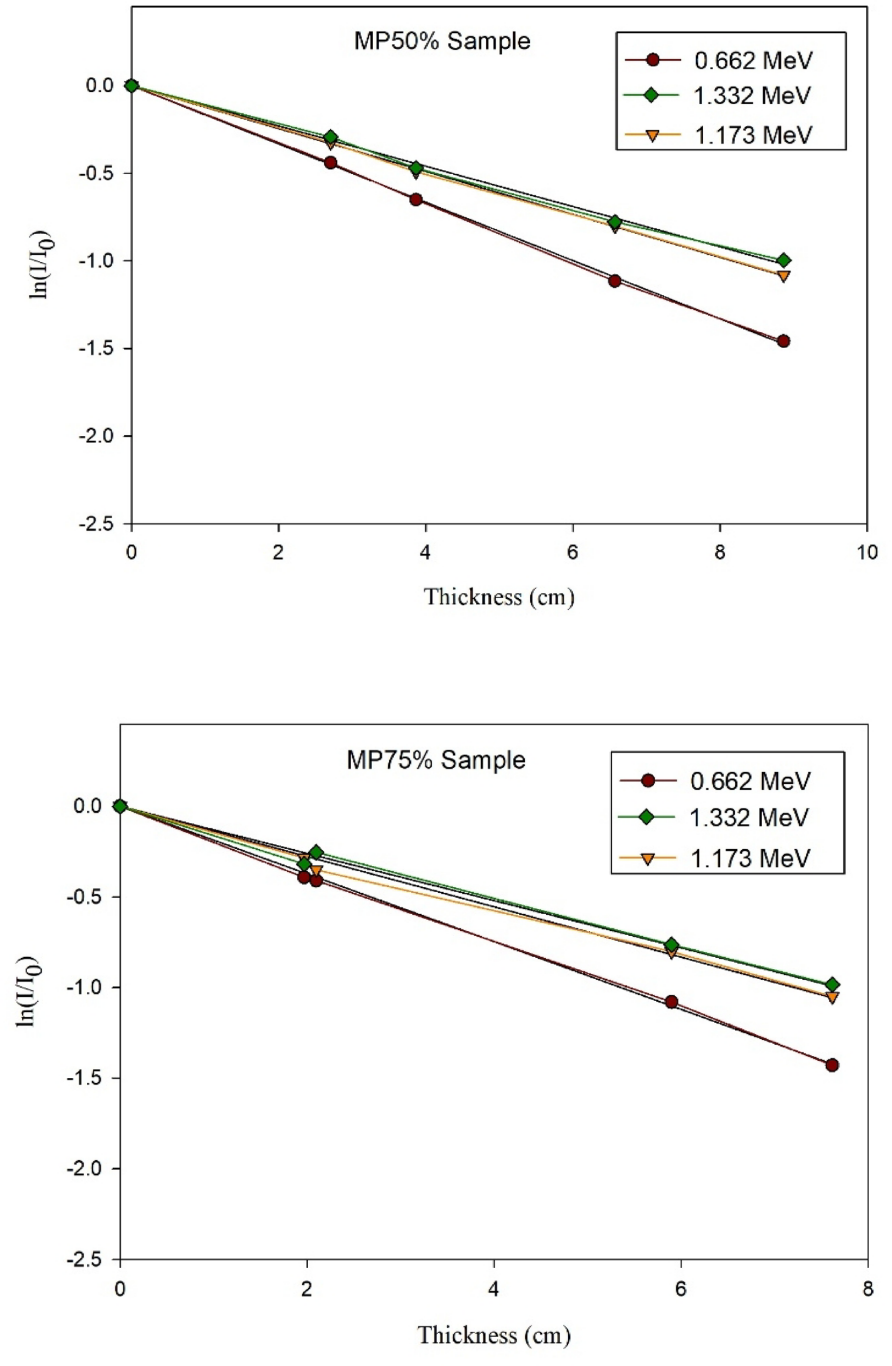

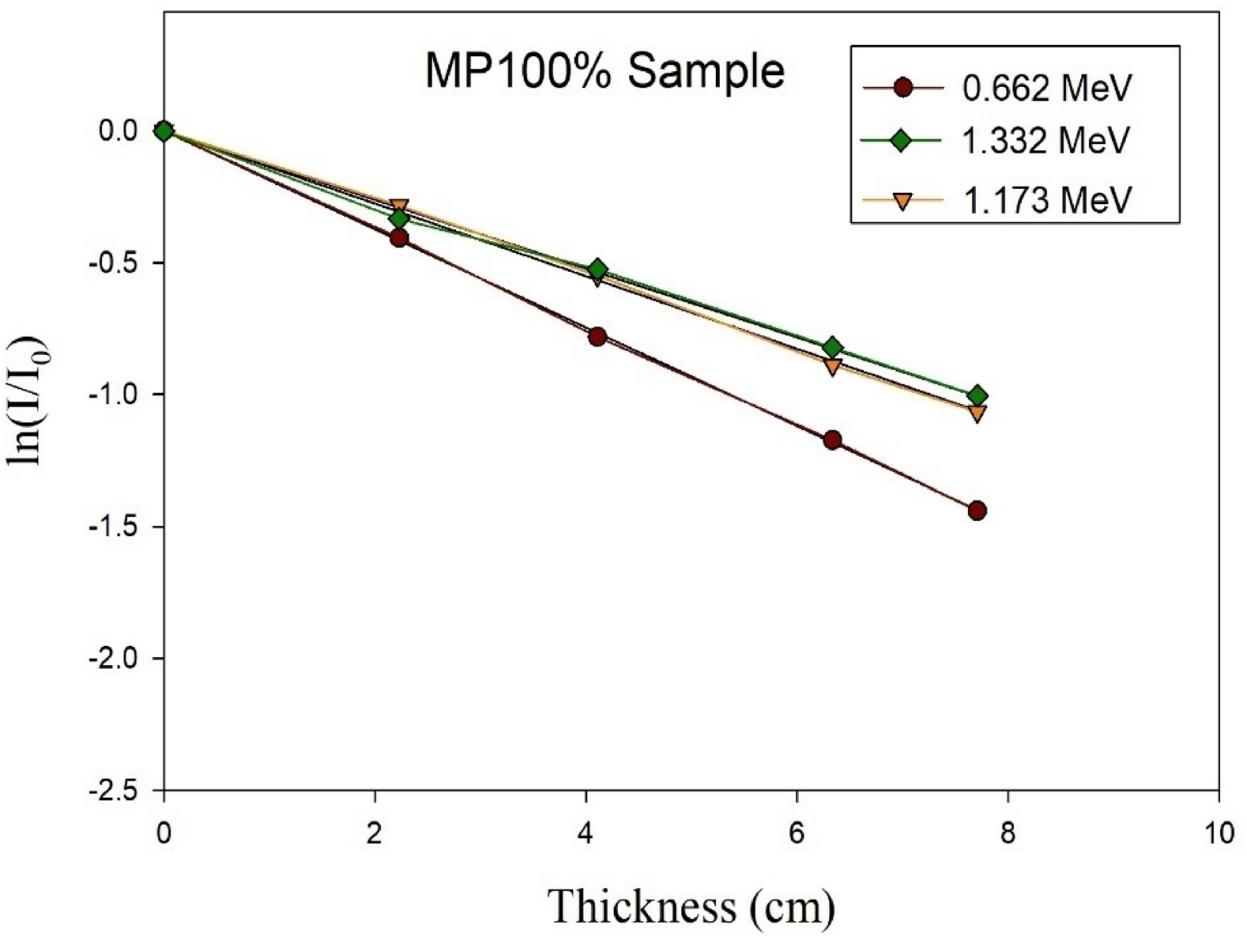
Experimental transmission curves of γ-rays relative intensity versus the thickness of cement plaster mixtures.

The absolute value of the slope of the optimally fitted curve has been taken as the linear attenuation coefficient at the energy of the corresponding photons. This conclusion was reached on the basis of the transmission curves that are shown in Fig. 10.

After that, the EpiXS software program was employed to compute the µ at the studied three gamma rays’ energies just for verification and to give the green light to proceed with the wider analytical investigation.

The experimentally and analytically obtained (µ) values are presented in Table 5 along with the calculated difference percentages to assess the agreement between the two calculating methods.

**Table 5:** Compatibility of experimental and analytical results

Mixture ID	LAC (cm^− 1^) at 0.661 MeV			LAC (cm^− 1^) at 1.173 MeV			LAC (cm^− 1^) at 1.333 MeV		
	EPIXS	Exp.	Percent Diff.	EPIXS	Exp.	Percent Diff.	EPIXS	Exp.	Percent Diff.
NP	0.177	0.161	9.47	0.134	0.122	9.37	0.126	0.121	4.05
MP25	0.171	0.166	2.97	0.139	0.133	4.41	0.13	0.124	4.72
MP50	0.197	0.181	8.47	0.150	0.147	2.02	0.140	0.128	8.96
MP75	0.202	0.192	5.08	0.153	0.153	0.00	0.143	0.133	7.25
MP100	0.209	0.195	6.93	0.158	0.157	0.63	0.148	0.138	6.99

Based on the obtained results shown in the former table, it could be observed the high compatibility between the experimentally obtained values and the analytically computed ones as the maximum percent difference detected was below 10% which proves the precission of the experimental setup used for the radiation attenuation measurements and also ensures the accuracy of the physicochemical characterizations of the prepared cement plasters. Also, increasing the magnetite content in the studied mixes shows increase in the value of the linear attenuation coefficient thus, improving the shielding capability of the cement plaster against the incident gamma rays. However, increasing the energy of the incident gamma radiation led to a notable decrease in the attenuation capability of the shielding cement plaster in general as the escaping probability of the incident photon increases due to the decrease in the interaction time with the shield constituents especially that the three studied energies are dominated by the Compton scattering mechanism which is the least dependent mechanism on the shield composition/effective atomic number.

### Analytical assessment of gamma rays’ shielding

**Fig. 11:**
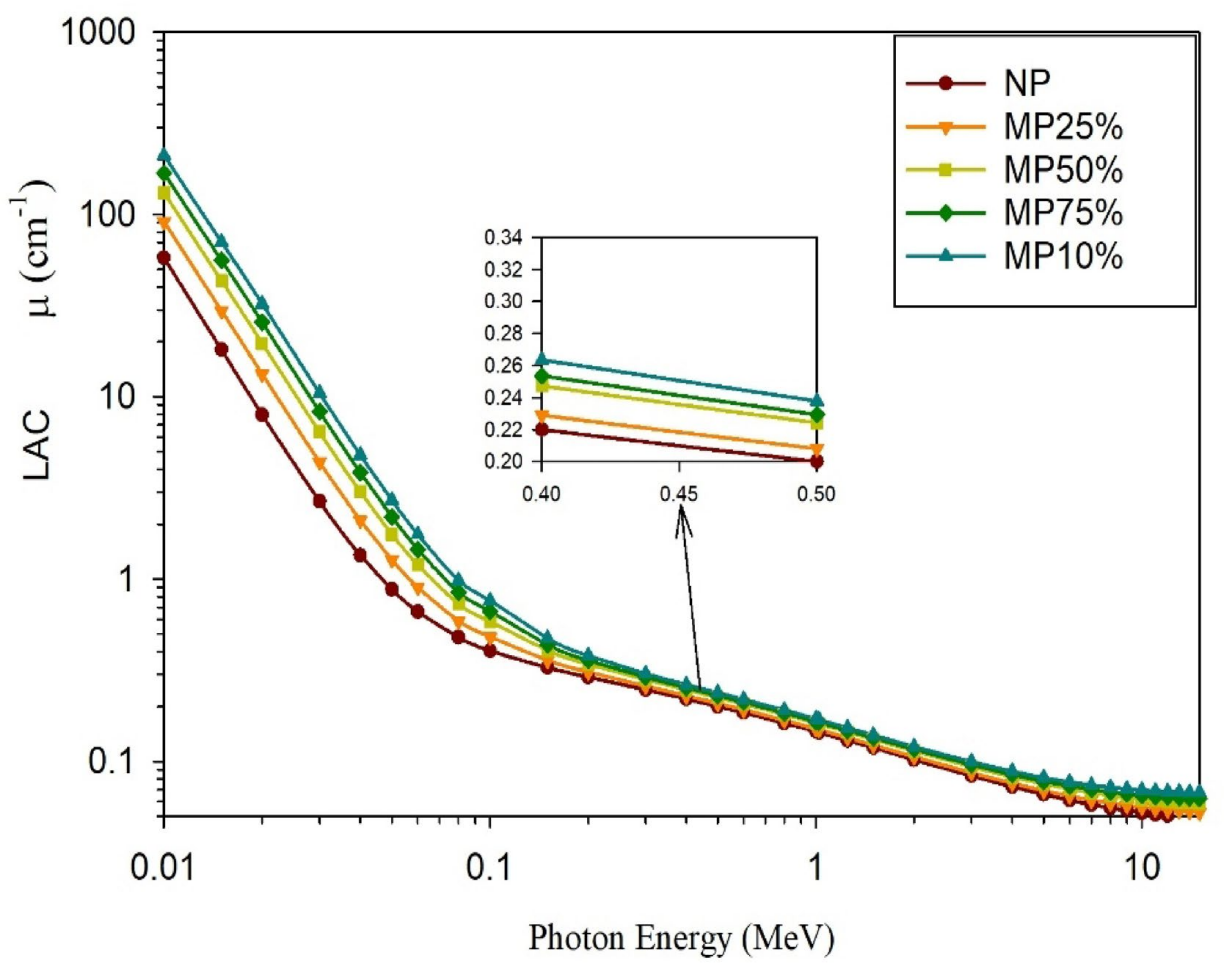
Evaluated LAC values for cement plaster mixtures using EpiXS software at the studied energy range.

**Fig. 12:**
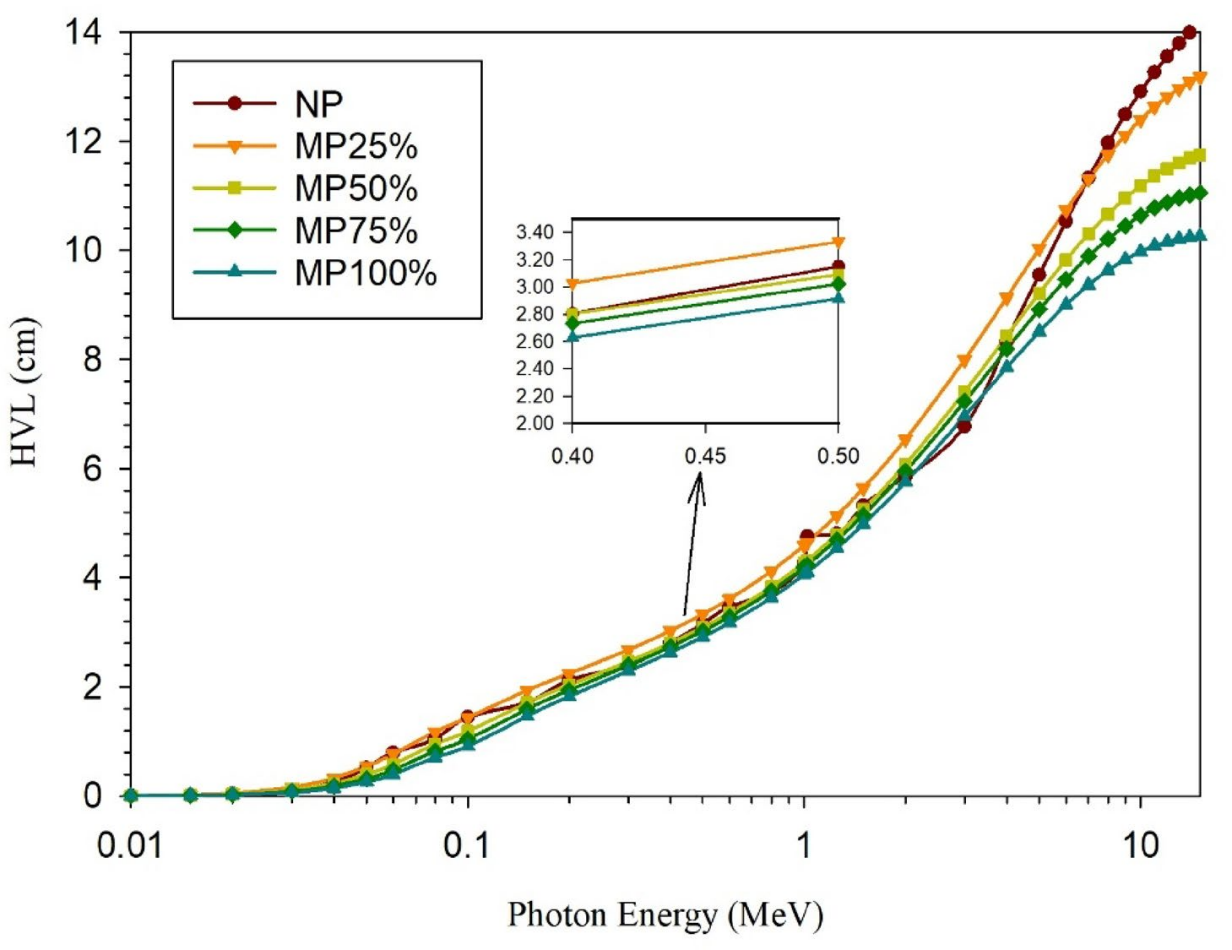
Evaluated HVL values for cement plaster mixtures using EpiXS software at the studied energy range.

**Fig. 13:**
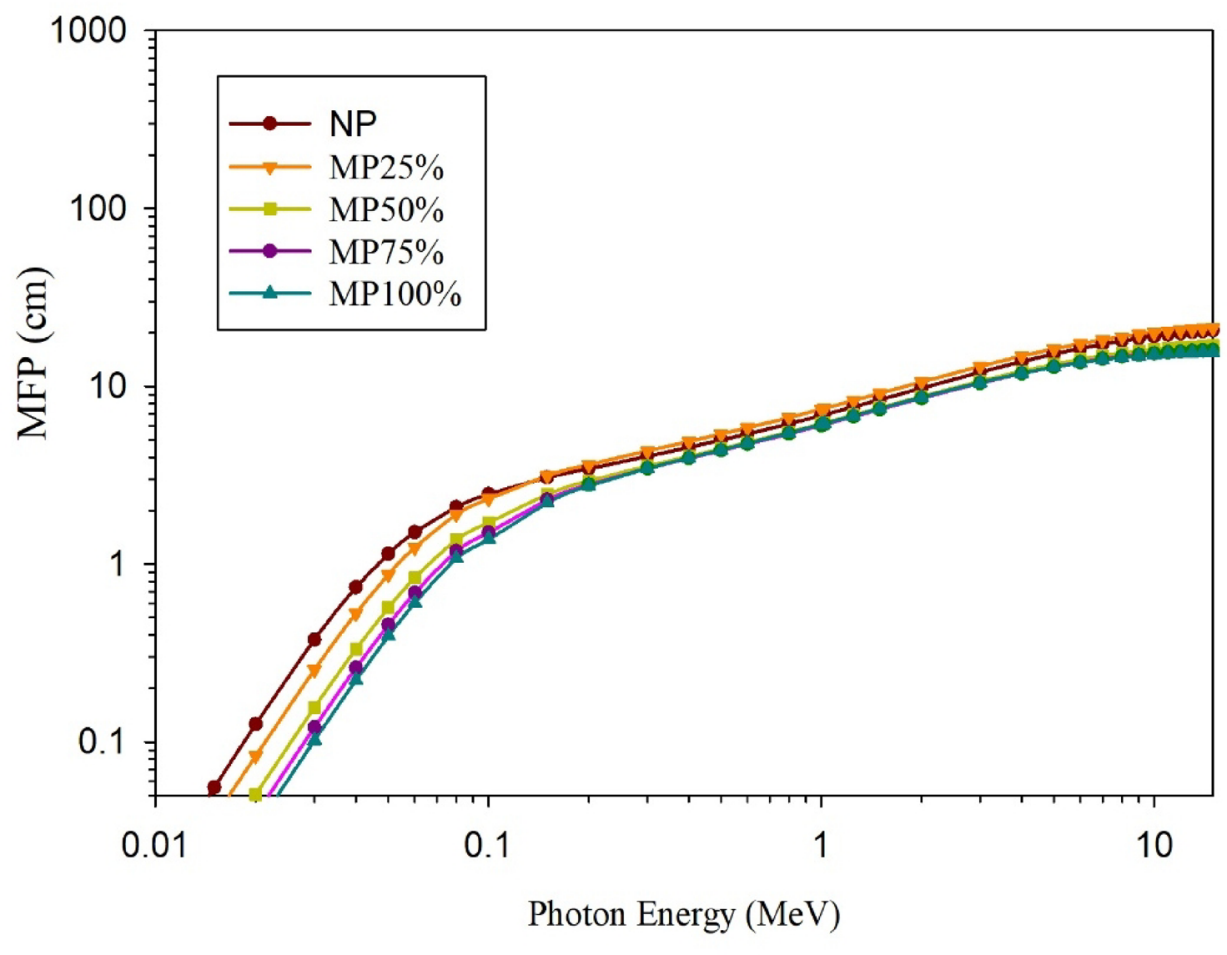
Evaluated MFP values for cement plaster mixtures using EpiXS software at the studied energy range.

**Fig. 14:**
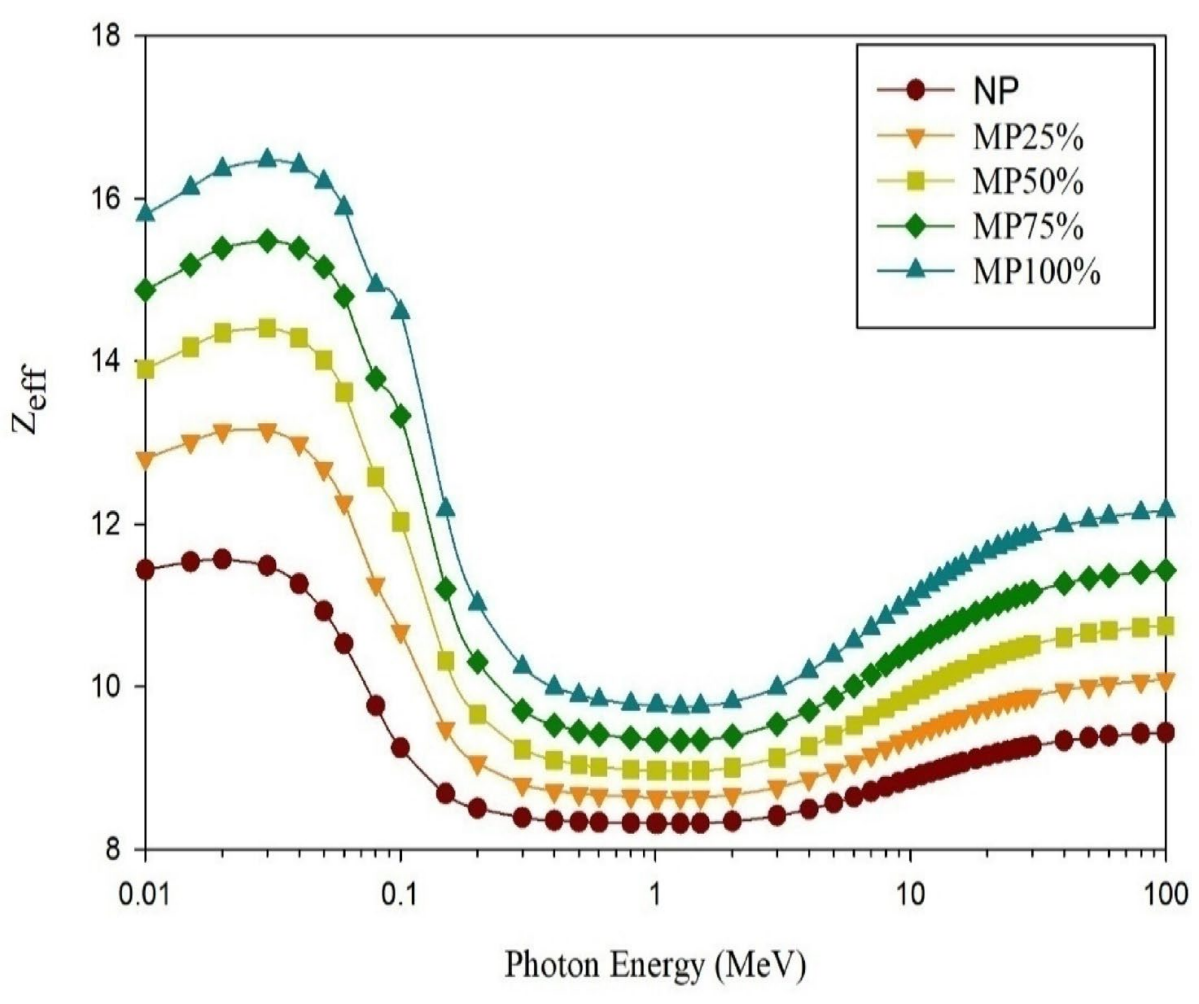
Evaluated Z_eff_ values for cement plaster mixtures using EpiXS software.

The computed gamma rays shielding parameters; µ, HVL, MFP, and Z_eff_, are shown in; Figs. 11, 12, 13, and 14, respectively.

Considering the gamma rays shielding properties of the studied samples, increasing the energy of the incident gamma radiation led to a decrease in the value of the computed µ for all samples. The µ drops from 57.8 to 0.047 cm^−1^ for (NP), 91.36 to 0.051 cm^−1^ for (MP25), from 131.4 to 0.058 cm^−1^ for (MP50), from 168.3 to 0.062 cm^−1^ for (MP75), and from 210.8 to 0.067 cm^−1^ for (MP100) at gamma rays’ energies range from 0.01 to 10 MeV. The rate of the mentioned decrease in µ values has been observed to be the greatest at low energies as the dominant mechanism at this energy range is the photoelectric mechanism which inversely correlates with Eγ^3.5^. Also the differences considering the linear attenuation coefficients are the biggest at this energy range as the photoelectric mechanism is in a direct proportionality with Z^4.5^ so, it significantly correlates with the effective atomic number of the studied cement plaster^24,34^. However, at the intermediate energy range, the aforementioned decreasing rate becomes lower and the differences among the studied cement plaster samples become smaller as the dominant mechanism is the Compton scattering mechanism whose dependency on the effective atomic number and the energy of the incident gamma rays is the least. A slight reincrease at the end of the studied energy range has been observed especially with increasing the magnetite content in the sample due to the increase in the contribution degree of the pair production mechanism which is proportional to the squared value of the atomic number of the attenuating medium. For all energies, especially at the lowest energies, it can be noted that increasing magnetite content increases the shielding effectiveness of the studied cement plaster as the constituent of the high-Z elements (Fe) increases which increases the effective atomic number of the studied cement plaster beside increasing the density.

It does make sense that both; HVL, which is the shield thickness required to attenuate half of the incident gamma rays, and the MFP, which is the average distance that can be traveled by the energetic photon in the attenuating medium without making any interaction, decrease with increasing the magnetite content in the sample due to the corresponding increase in the shielding efficiency.

The experimental findings obtained in this study were compared with a wide range of previously published research to validate the observed trends and place the results within the broader scientific context. The reduction in workability associated with increasing magnetite content aligns with the observations reported by Sikora et al.^13^, who demonstrated that incorporating Fe_3_O_4_ nanoparticles increases viscosity and reduces early-age flowability due to the higher specific surface area of magnetite particles. Similarly, the notable increase in the density of the prepared plasters is consistent with earlier investigations on magnetite- and heavyweight-aggregate-based composites, where magnetite was shown to significantly enhance unit weight and consequently improve radiation attenuation performance^6,17,18^. Regarding compressive strength, the decreasing trend recorded for mixtures with higher magnetite replacement agrees with findings by Ali et al.^15^ and Ghorbani et al.^6^, who attributed strength reduction to lower compactness and less efficient particle packing in magnetite-rich matrices. Despite this reduction, the obtained strengths remain within acceptable limits for non-load-bearing shielding applications, which supports similar recommendations in the literature for heavy or radiation-protective coatings. The γ-ray attenuation results also follow expected behavior and support conclusions from previous studies, where magnetite, hematite, and other iron-rich minerals were shown to markedly enhance linear attenuation coefficients, particularly at low photon energies dominated by the photoelectric effect^4,6^. The superior shielding efficiency of the MP100 mixture corresponds with the strong correlation between effective atomic number, material density, and photon interaction probability, as confirmed by EpiXS-based analytical assessments and previously reported datasets^23,27^.

### Analytical assessment of fast neutrons’ shielding

**Fig. 15:**
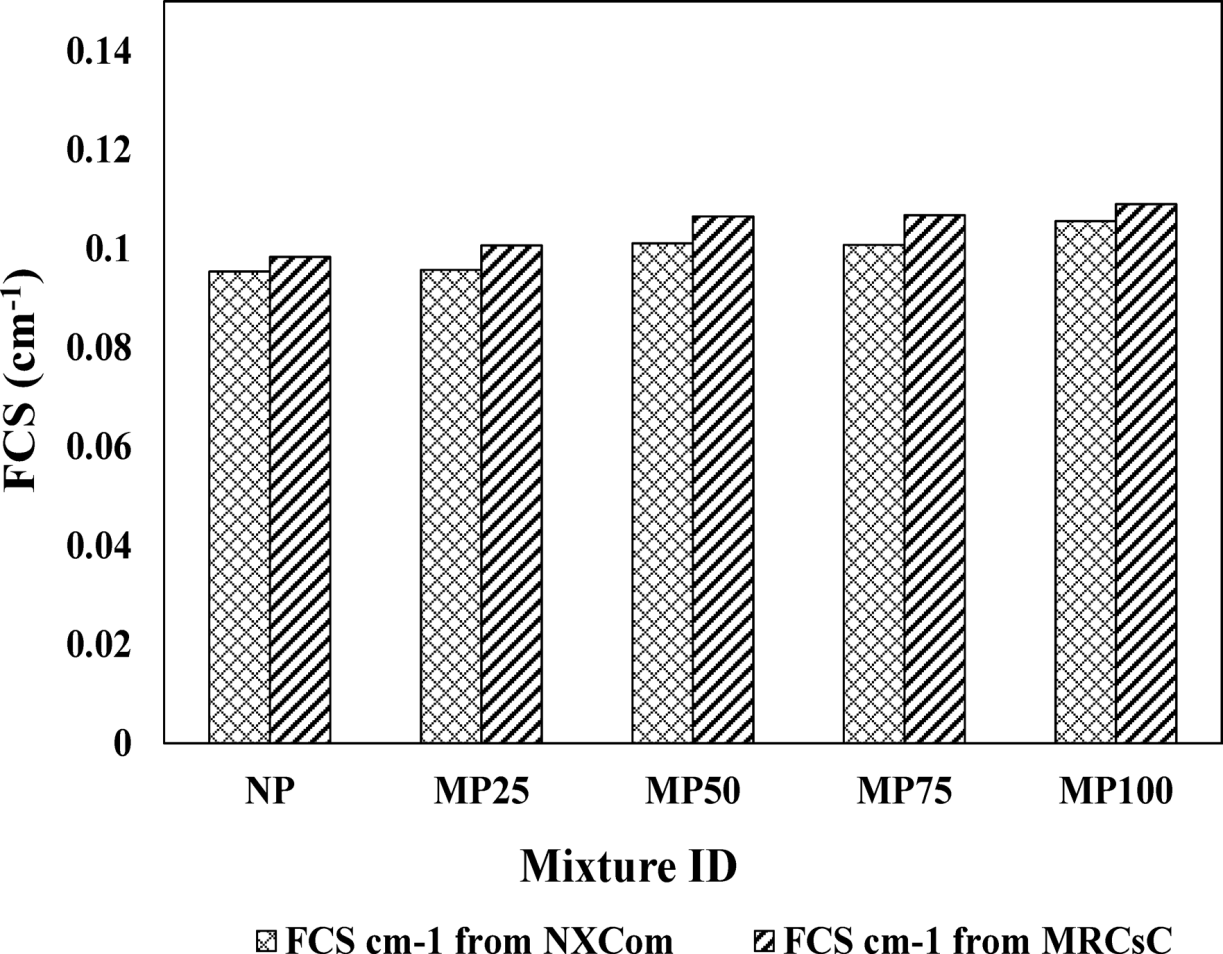
NXCom and MRCsC results for all cement plaster mixtures.

The macroscopic fast neutrons removal cross-section (FCS) was computed for all prepared cement plaster samples employing NXCom and MRCsC software programs as shown in Fig. 15; Table 6. The MP100 sample was found having the highest value for FCS (0.1073 cm^− 1^) compared to the other prepared samples, and thus, the lowest value for the relaxation length (λ) (93.2 mm). Considering that almost all prepared mixes have the same water and light elements’ content, the possible reason for the superior fast neutrons attenuation capability of MP100 plaster sample is that it has the highest magnetite content thus, the highest content of iron compared to the other mixes along with the highest density. Iron has notable microscopic fast neutrons removal cross-section compared to the other intermediate and high-Z elements due to its appreciated inelastic scattering cross-section as a primary mode of interaction with the incident fast neutrons. Also, based on the linear summation rule that was discussed before, there is a direct correlation between FCS and the density of the shield as higher density of the shield means higher nuclear density thus, increase in the interaction probability between the incident energetic neutron and the shield.

For fast neutron shielding, the enhanced macroscopic removal cross-sections recorded for magnetite-rich mixtures agree with the well-established neutron-moderating role of iron and its substantial inelastic scattering cross-section, a trend further supported by NXCom and MRCsC predictions. This behavior is consistent with earlier studies on heavy concrete mixes containing magnetite and other high-Z aggregates^23,31^, confirming that magnetite incorporation significantly improves both photon and fast neutron attenuation. Overall, the alignment of the present results with published literature reinforces the validity of the experimental outcomes and highlights the potential of magnetite-based plaster as an efficient composite for radiation shielding applications in nuclear infrastructure.

**Table 6:** Evaluated FCS for cement plaster Mixtures using NXCom and MRCsC softwares.

Mixture ID	FCS (cm^− 1^)		Avg. value
	NXCom (lower limit)	MRCsC (upper limit)	
NP	0.0953	0.0983	0.0968
MP25	0.0957	0.1006	0.0982
MP50	0.1010	0.1065	0.1037
MP75	0.1017	0.1068	0.1038
MP100	0.1056	0.1090	0.1073

## Conclusions

This study establishes a foundation for a practical use of magnetite-cement plaster in the maintenance and repair of nuclear power plant structures, particularly in repair or retrofitting scenarios where conventional cement plaster is insufficient. The investigated parameter was the feasibility and effectiveness of using magnetite powder as a partial or full replacement for natural silica sand in cement plaster manufacturing. A series of experimental and analytical evaluations were conducted to examine the mechanical and radiation shielding properties of the proposed cement plaster mixtures, supported by software-based calculations using EpiXS, NXCom, and MRCsC. The conclusion was drawn as follows:

- The flowability of the cement plaster matrix was generally decreased by up to 46.2% due to implementing the magnetite powders in its production. This can lead to less effective compactness, therefore affecting the mechanical properties.
- The MP100 mixture achieved the highest density (48.7% higher than normal cement plaster) among all other mixtures owing to full replacement of silica sand with magnetite powder. This directly correlated with enhanced radiation attenuation performance.
- Generally, the compressive strength of cement plaster mixtures decreased by up to 65.4% as magnetite content increased, which could be attributed to the decreased compactness and packing density.
- Results from experimental gamma-ray attenuation tests showed excellent agreement with the analytical predictions from EpiXS, with percentage differences generally within acceptable ranges (below 10%). The combination of experimental validation and software analysis confirmed the reliability of magnetite-based cement plasters, considering both photon and fast neutron shielding.
- Replacing natural sand with magnetite significantly enhances the radiation shielding properties of cement plaster, with the MP100 mixture (100% magnetite) showing the best performance in both gamma-ray and fast neutron attenuation. The MP100 mixture demonstrated the highest linear attenuation coefficient (LAC) for gamma rays (264% increase at 0.01 MeV and 43% increase at 10 MeV compared to the traditional mix) and the best fast neutron shielding performance, with a macroscopic removal cross-section (Σ_R_) of 0.1056 cm⁻¹ (11% increase compared to the traditional mix).
- Magnetite’s higher density and iron content reduced the mean free path of gamma rays, increased the neutron scattering and improved the shielding efficiency against both energetic photons and fast neutrons.
- For optimal mix selection where mechanical strength is critical alongside shielding, a partial replacement ratio of silica sand with magnetite powder by 75% (MP75) provides a good balance between mechanical properties and radiation shielding efficiency since the full replacement (100%) didn’t achieve the structural adequacy.

Finally, this study paves the way for more research on the feasibility of producing non-traditional cement plaster mixtures with enhanced radiation-shielding performance for structural repairs or reinforcement of shielding walls in critical facilities.

## Data Availability

All data generated or analyzed during this study are included in this published article.
